# Immunohistochemical molecular phenotypes of gastric cancer based on SOX2 and CDX2 predict patient outcome

**DOI:** 10.1186/1471-2407-14-753

**Published:** 2014-10-09

**Authors:** Vânia Camilo, Rita Barros, Ricardo Celestino, Patrícia Castro, Joana Vieira, Manuel R Teixeira, Fátima Carneiro, João Pinto-de-Sousa, Leonor David, Raquel Almeida

**Affiliations:** Institute of Molecular Pathology and Immunology of the University of Porto (IPATIMUP), Rua Dr. Roberto Frias s/n, 4200-465 Porto, Portugal; School of Allied Health Sciences ESTSP, Polytechnic of Porto, Porto, Portugal; Department of Genetics, Portuguese Oncology Institute, Porto, Portugal; Abel Salazar Biomedical Sciences Institute, University of Porto, Porto, Portugal; Department of Pathology and Oncology, Faculty of Medicine of the University of Porto, Porto, Portugal; Department of Pathology, Centro Hospital S. João, Porto, Portugal; Department of Surgery, Faculty of Medicine of the University of Porto, Porto, Portugal; Department of Biology, Faculty of Sciences of the University of Porto, Porto, Portugal

**Keywords:** SOX2, CDX2, Gastric cancer, Prognosis, Survival

## Abstract

**Background:**

Gastric cancer remains a serious health concern worldwide. Patients would greatly benefit from the discovery of new biomarkers that predict outcome more accurately and allow better treatment and follow-up decisions. Here, we used a retrospective, observational study to assess the expression and prognostic value of the transcription factors SOX2 and CDX2 in gastric cancer.

**Methods:**

SOX2, CDX2, MUC5AC and MUC2 expression were assessed in 201 gastric tumors by immunohistochemistry. SOX2 and CDX2 expression were crossed with clinicopathological and follow-up data to determine their impact on tumor behavior and outcome. Moreover, *SOX2* locus copy number status was assessed by FISH (N = 21) and Copy Number Variation Assay (N = 62).

**Results:**

SOX2 was expressed in 52% of the gastric tumors and was significantly associated with male gender, T stage and N stage. Moreover, SOX2 expression predicted poorer patient survival, and the combination with CDX2 defined two molecular phenotypes, SOX2^+^CDX2^-^ versus SOX2^-^CDX2^+^, that predict the worst and the best long-term patients’ outcome. These profiles combined with clinicopathological parameters stratify the prognosis of patients with intestinal and expanding tumors and in those without signs of venous invasion. Finally, *SOX2* locus copy number gains were found in 93% of the samples reaching the amplification threshold in 14% and significantly associating with protein expression.

**Conclusions:**

We showed, for the first time, that SOX2 combined with CDX2 expression profile in gastric cancer segregate patients into different prognostic groups, complementing the clinicopathological information. We further demonstrate a molecular mechanism for SOX2 expression in a subset of gastric cancer cases.

**Electronic supplementary material:**

The online version of this article (doi:10.1186/1471-2407-14-753) contains supplementary material, which is available to authorized users.

## Background

Gastric cancer is one of the most commonly diagnosed cancers and now the third leading cause of cancer-related deaths in the world [1]. Laurén classification divides gastric cancer into two main histological types: intestinal and diffuse [2]. They present distinct morphological, clinical and epidemiological features and are thought to develop from the activation of independent molecular mechanisms. Epidemiological data shows that, in most cases, gastric cancer does not arise *de novo* from the normal gastric epithelium, but rather results from a multistep process, through successive genetic and epigenetic alterations in multiple genes. Contrary to the diffuse type, for which predisposing lesions are not well established, the succession of events leading to gastric cancer of the intestinal type is well described. According to the Correa’s cascade, it develops in a stepwise manner, usually initiated by an inflammatory process triggered by *Helicobacter pylori*, which may progress to multifocal atrophic gastritis, intestinal metaplasia (IM), dysplasia and finally adenocarcinoma [3].

Even though there has been a steady decline in gastric cancer incidence globally, the expansion and aging of the World’s population forecasts an increase in the number of cases and, despite the latest developments in diagnosis and treatment, the prognosis of gastric cancer patients remains poor. This translates into a 5-year survival rate of no more than 25%, in Europe [4]. Most gastric cancer patients have advanced disease at diagnosis for whom the only option of cure relies on complete surgical removal of the tumor, with extensive lymph node dissection that can be complemented by the administration of neoadjuvant or adjuvant chemotherapy [5]. The most important prognostic factor influencing survival as well as treatment choice is the TNM stage. However, an additional layer of complexity stems from the observation that patients with the same staging often show a different clinical evolution, highlighting the heterogeneity of gastric cancer [6]. This suggests that unique intrinsic biological properties of these tumors may significantly impact their aggressive potential. Therefore, there is still a pressing need to discover additional prognostic biomarkers to predict outcome more accurately and, as a result, to help make better informed treatment decisions with respect to therapy. As tumors that are more differentiated generally behave in a less aggressive fashion, we questioned the relevance of two differentiation markers, SOX2 and CDX2, for the biological behavior of gastric cancers. These markers are differentially expressed along the chain of events that lead to gastric cancer and are closely associated with gastric and intestinal differentiation, respectively [7].

CDX2 is an intestine-specific homeobox transcription factor whose function ensures the development and maintenance of intestinal differentiation in the gut and ectopic sites, which was well established in animal models [8, 9]. It is widely known that CDX2 is expressed in human intestinal metaplasia of the stomach and esophagus [10]. It was shown to be also expressed in dysplasia thus reinforcing its role as a biomarker of progression in the preneoplastic stages of gastric carcinogenesis [11].

SOX2 is a member of the SOX (SRY-related HMG Box) family of transcription factors, encoded by a highly conserved, single exon gene. SOX2 plays diverse roles throughout development and cell differentiation, first orchestrating the mammalian embryogenesis [12], and later contributing to the normal morphogenesis and homeostasis of the foregut-derived epithelia of the esophagus, lung and trachea [13]. In adulthood, SOX2 is expressed in a variety of tissues, namely in the squamous epithelium lining the esophagus and the glandular epithelium of the stomach. It has been shown, in mice, that Sox2 expression contributes to all the cell lineages normally found in the stomach, suggesting an important contribution for gastric differentiation [14]. In addition, abnormal expression of SOX2 has been observed in tumors of the brain, breast, lung and esophagus [15–17]. However, in the gastric cancer context, its role remains puzzling and needs further clarification [18–20]. Furthermore, its interplay with CDX2 remains unexplored.

Our aim was to analyze the clinical relevance of SOX2 and CDX2 expression in gastric cancer biology. The results obtained show that their expression in the primary tumors has a relevant prognostic value for gastric cancer patients.

## Methods

### Human tissues and DNA samples

Two hundred and one cases of formalin-fixed paraffin-embedded (FFPE) samples from patients with gastric adenocarcinoma undergoing surgery at Centro Hospitalar S. João, Porto, Portugal between 1988 and 2010 were studied. Cases selected were those with available paraffin-embedded material, clinicopathological and follow-up data obtained from the Pathology and Surgical Departments of Centro Hospitalar S. João. From those, sixty-seven samples were provided as a tissue microarray (TMA), with 2 replicate cores of each sample, from the Tumor and Tissue Biobank of the same Pathology Department. Tumour genomic DNA (gDNA) was obtained from 62 cases included on the TMA. gDNA was also extracted from FFPE samples of two normal gastric mucosas using the Genomic DNA Purification Kit (Citomed, Lisbon, Portugal), and used as controls. All samples from the Biobank were obtained with informed consent from the patients. The use of retrospective samples from which informed consent cannot be obtained is authorized for research studies by the Portuguese law. This study was approved by the ethics committee of Centro Hospitalar S. João.

### Immunohistochemistry

FFPE tissue sections with 4 μm from surgical specimens and TMA were subjected to immunohistochemistry for SOX2, CDX2, MUC5AC and MUC2, following standard methodologies. Briefly, after deparaffination and rehydration, antigen retrieval was performed in a IHC-Tek Epitope Retrieval Steamer Set (IHC World, Woodstock, MD, USA), for 40 minutes with 10 mM citrate buffer, pH 6.0 (CDX2) or 10 mM, pH 8.0 EDTA (SOX2). Desialylation was performed for MUC2 detection, with 0.1 U/mL of Neuraminidase from *Clostridium perfringes* type VI (Sigma-Aldrich, St. Louis, MO, USA) in sodium acetate buffer pH 5.5 for 2h at 37°C. Endogenous peroxidase was blocked with 3% hydrogen peroxide in methanol for 10min. Incubation with primary antibodies for CDX2 (1:50 dilution, CDX2-88 clone, Biogenex, San Ramon, CA, USA), MUC5AC (1:10 dilution, CLH2 clone) and MUC2 (1:10 dilution, PMH1 clone) was performed overnight, at 4°C. Sections were then incubated with a biotin-labeled rabbit anti-mouse secondary antibody, followed by the avidin/biotin-peroxidase detection system (Vectastain ABC kit, Vector Laboratories, Burlingame, CA, USA). For SOX2 staining, incubation with the primary antibody (1:50 dilution, SP76 clone, Cell Marque, Rockling, CA, USA) was performed for 1h at RT and detection was done using the Dako REAL™ Envision™ Detection System Peroxidase/DAB + (DAKO, Glostrup, Denmark) according to the manufacturer’s instructions. Detection of expression was performed with 3,3′-diaminobenzidine (DAB) (Sigma,St. Louis, MO, USA) tissue sections were counterstained with Gill’s haematoxylin (Leica Microsystems, Amersham, Bucks, UK), dehydrated, clarified and mounted. Normal gastric mucosa was used as a positive control for SOX2 and MUC5AC expression and normal colonic mucosa was used as a positive control for CDX2 and MUC2 expression. Cases were considered positive when more than 5% of the cells were stained with each antibody with consensus of three observers (VC; LD and RA).

### Fluorescence *In Situ*Hybridization (FISH)

FISH was used to assess *SOX2* amplification status in 21 samples, using a 2-color assay as described by Bass *et al* [17]. A probe spanning the locus 3q26.33 (BAC clone CTD-2348H10) was used to determine *SOX2* copy number and was compared with a reference probe hybridizing to 3p22.3-3p22.2 (BAC clone RP11-286G5), purchased from Invitrogen (Carlsbad, CA, USA). The target probe was labelled with digoxigenin (DIG-11-UTP, Roche, Mannheim, Germany) and detected with an anti-digoxigenin–fluorescein antibody (Roche Diagnostics GMbH, Mannheim, Germany) and the reference probe was labelled with biotin (BioPrime® DNA labelling System, Invitrogen, Carlsbad, CA, USA) and detected with a CY_3_-Avidin antibody (Jackson Immunoresearch Lab, West Grove, USA).

Four μm slides from each gastric adenocarcinoma sample were deparaffinised, rehydrated and placed in a pre-heated solution of NaSCN 1M at 80°C for 10 min. Samples were digested with 6mg/ml pepsin (Sigma-Aldrich, St. Louis, MO, USA) in HCl 0.02N, pH 2 for 30min at 37°C. Probe mixture in 50% formamide in 2× SSC was codenatured with nuclear DNA at 94°C for 3min. Hybridisation was carried out for 30h at 37°C. Nuclei were counterstained with DAPI-Vectashield mounting solution (Vector, Burlingame, USA). For each case, a minimum of 67 cells were analysed in a fluorescence microscope (Zeiss Z1 Axio) and images were acquired with a 1000× magnification, using a Zeiss Axio cam MRm and the Axiovision Rel. 4.8 software. The ratio between the number of SOX2 and the reference probe signals was calculated for each cell individually and the average of the ratios was determined for each case. Gene amplification was considered whenever the average was ≥2.

### Copy Number Variation (CNV) Assays

The CNV assay was performed using a specific TaqMan® Copy Number Assay (Applied Biosystems, Foster City, CA, USA) following the manufacturer’s instructions. Briefly, multiplex PCRs for *SOX2* (target) and *Rnase P* (endogenous control) were performed on an ABI Prism7500 Fast Real-time PCR system using the software v2.0 (Applied Biosystems, Foster City, CA, USA). Twenty nanograms of gDNA and a non-template control were analyzed in quadruplicate. Normal gastric mucosa samples were used as calibrators. Quantification of *SOX2* gene copy number was done using the ΔΔCt method and data analysis was performed by the CopyCaller software v2.0 (Applied Biosystems, Foster City, CA, USA). Gene amplification was defined whenever the gene copy number was 2-fold greater than that of the normal gastric mucosa. Fold increase between 1.5 and 2 was considered copy number gain.

### Statistical analysis

The chi-square test was used to assess the significance of differences in clinicopathological characteristics across categories of SOX2 and CDX2 expression, except with age, where the significance of differences in means was calculated using the *T*-test. Patients’ overall survival was calculated using the Kaplan-Meier method and the significance of differences between crude survival curves was tested by the log-rank test. Cox proportional hazards model was used to calculate univariate and multivariate hazard ratios. Median follow-up time for surviving patients was 62 months. The 5- and 10-year survival rate was estimated using the life-table method. Differences were considered statistical significant whenever the *p* values were <0.05. Analyses were performed using SPSS software v21.0 (Chicago, IL, USA) and Stata v11 (College Station, TX, USA).

## Results

### SOX2 expression in gastric carcinomas

**Figure 1:**
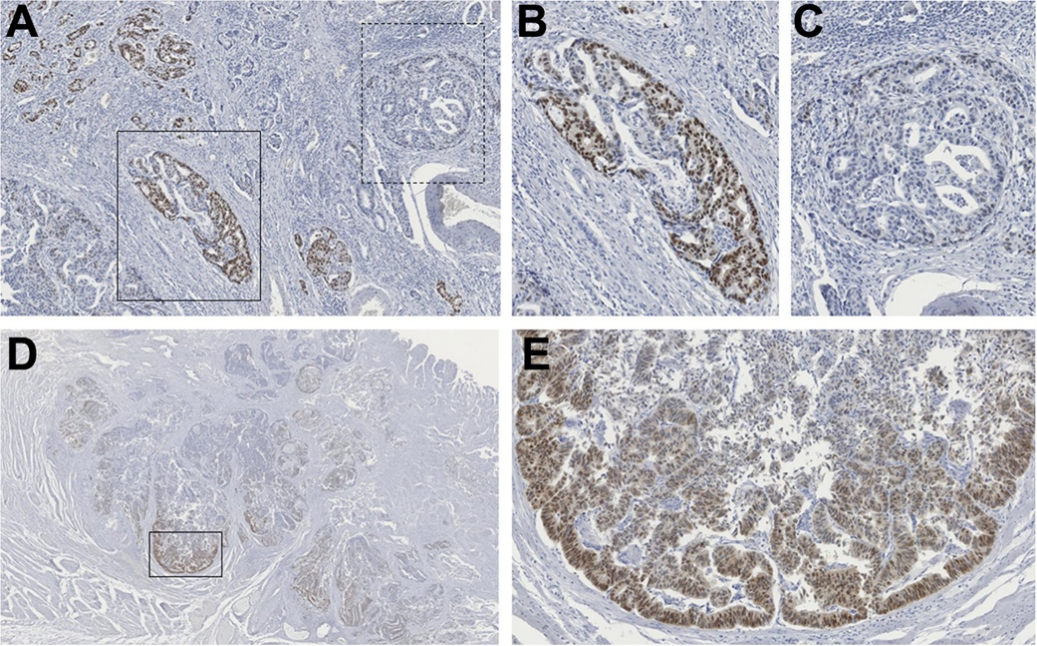
**SOX2 protein expression in gastric carcinomas, detected by immunohistochemistry. (A)** Heterogeneity of SOX2 expression in gastric tumors. Within the same tumor sample, areas with high levels of SOX2 are observed **(B)** in close proximity to areas with lower expression levels of SOX2 **(C)**. **(D, E)** SOX2 expression at the invasive front of tumors.

Clinicopathological features of the 201 gastric cancer cases, as well as the expression profiles of SOX2, CDX2, MUC2 and MUC5AC are summarized in Table 1. Nuclear SOX2 expression was found in 52% (104/201) of gastric carcinomas and the expression was heterogeneous among and within the tumors (Figure 1A, B and C). In 13% (18/134) of the cases SOX2 expression was higher at the invasive front (Figure 1D and E) (this evaluation was only performed in cases not included in the TMA, where this assessment was not possible).

**Table 1:** **Summary of the clinicopathological parameters of gastric cancer according to the expression of SOX2 and CDX2**

	SOX2					CDX2				
	Negative		Positive			Negative		Positive		
	N	%	N	%	p	N	%	N	%	p
Age, years^1^										
Mean ± SD	65.8 ± 12.7		63.4 ± 13.5		0.2	64.1 ± 13.6		65.2 ± 12.6		0.6
Median (Range)	69 (34-85)		66.5 (24-89)			69 (24-89)		67 (36-89)		
Sex^1^										
Male	52	41.9	72	58.1	0.02	75	60.5	49	39.5	0.1
Female	45	58.4	32	41.6		38	49.4	39	50.6	
TNM stage^2^										
I/II	35	54.7	29	45.3	0.09	26	40.6	38	59.4	0.002
III/IV	59	44.7	73	55.3		83	62.9	49	37.1	
T stage^3^										
T1/T2	22	62.9	13	37.1	0.03	13	37.1	22	62.9	0.007
T3/T4	75	45.5	90	54.5		99	60.0	66	40.0	
N stage^1^										
N0	28	62.2	17	37.8	0.03	20	44.4	25	55.6	0.07
N+	69	44.2	87	55.8		93	59.6	63	40.4	
Margins^4^										
R0	85	47.5	94	52.5	0.5	98	54.7	81	45.3	0.2
R1	11	55.0	9	45.0		14	70.0	6	30.0	
Vascular invasion^4^										
Negative	39	55.7	31	44.3	0.1	37	52.9	33	47.1	0.5
Positive	57	44.2	72	55.8		75	58.1	54	41.9	
Láuren^5^										
Intestinal	44	49.4	45	50.6	0.9	45	50.6	44	49.4	0.3
Diffuse	20	48.8	21	51.2		26	63.4	15	36.6	
Unclassified	31	46.3	36	53.7		40	59.7	27	40.3	
Ming^6^										
Expansive	32	53.3	28	46.7	0.5	28	46.7	32	53.3	0.1
Infiltrative	60	45.8	71	54.2		81	61.8	50	38.2	
Unclassified	2	66.7	1	33.3		1	33.3	2	66.7	
SOX2^1^										
Negative						58	59.8	39	40.2	0.3
Positive						55	52.9	49	47.1	
CDX2^1^										
Negative	58	51.3	55	48.7	0.3					
Positive	39	44.3	49	55.7						
MUC5AC^1^										
Negative	39	44.8	48	55.2	0.4	42	48.3	45	51.7	0.05
Positive	58	51.3	55	48.7		70	61.9	43	38.1	
MUC2^1^										
Negative	69	48.6	73	51.4	0.9	84	52.2	58	40.8	0.1
Positive	27	48.2	29	51.8		26	46.4	30	53.6	

^1^N = 201; ^2^N = 196; ^3^N = 200; ^4^N = 199; ^5^N = 197; ^6^N = 194.

Next, we assessed whether SOX2 expression correlated with clinicopathological features of the cases. This analysis revealed that SOX2 expression was significantly associated with male gender (*p* = 0.02), with higher T stage (*p* = 0.03) and with presence of regional lymph node metastases (N stage) (*p* = 0.03). The other clinicopathological parameters, which included age, TNM stage, tumor clearance at resection margins (R category), vascular invasion, Laurén classification and Ming classification were not significantly associated with SOX2 expression (Table 1).

Since SOX2 is associated with gastric differentiation [14] we studied whether this was maintained in cancer, using MUC5AC as a gastric marker. However, no association was found between SOX2 and MUC5AC expression. Likewise, no reverse association was found between SOX2 and CDX2/MUC2 (used as markers of intestinal differentiation). Of note, CDX2 expression was significantly associated with a lower TNM stage (*p* = 0.002) and with a lower T stage (*p* = 0.007). Furthermore, although not statistically significant, there was a trend towards an inverse relationship between CDX2 and MUC5AC (*p =* 0.05).

### Survival analysis

**Figure 2:**
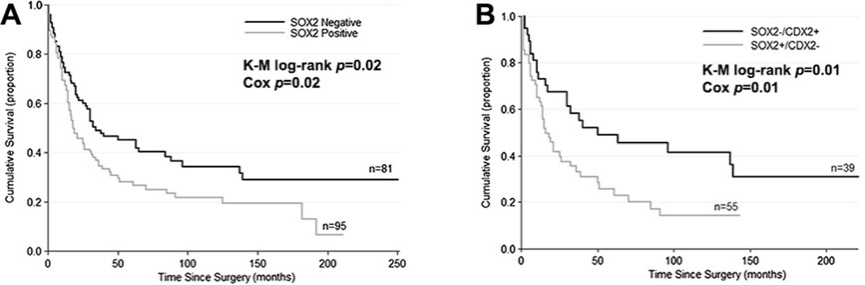
**Kaplan-Meier curves showing the probability of overall survival for patients with gastric cancer according to SOX2 expression (A) and according to the molecular phenotypes SOX2** ^**-**^ **/CDX2** ^**+**^ **and SOX2** ^**+**^ **/CDX2** ^**-**^ **(B).**

The 5-year overall survival, based on the follow-up of 199 patients, was 35% (Additional file 1: Figure S1). We next observed that SOX2 expression in the tumors was associated, in univariate analysis, with an overall poorer survival (*p* = 0.02), stratifying the patients into two prognostic groups (Figure 2A), with a 5-year survival of 45% for patients with SOX2- tumors versus 26% for patients with SOX2+ tumors. On the contrary, CDX2 expression associated with an overall improved survival, although the difference did not reach significance (data not shown).

Based upon the association of SOX2 and CDX2 with worse and better survival as well as more and less aggressive clinicopathological features, respectively, we further studied patients’ outcome according to the molecular phenotypes defined by both proteins. Four groups of patients could be defined (Additional file 1: Figure S2) of which the one with the best survival had tumors negative for SOX2 and positive for CDX2 (SOX2-/CDX2+), whereas the survival was the lowest in patients harboring tumors with the reverse profile, positive for SOX2 and negative for CDX2 (SOX2+/CDX2-) (*p* = 0.01) (Additional file 1: Figure S2 and Figure 2B). The cases with expression of both SOX2 and CDX2 or without expression of either marker exhibited a similar and intermediate survival (Additional file 1: Figure S2). The SOX2-/CDX2+ and SOX2+/CDX2- molecular phenotypes, compared with SOX2 alone, predict very similar survival rates at 5-years (49% vs 26% and 45% vs 26%, respectively). However, the predicted 10-year survival rate based on SOX2 expression alone is 34% (SOX2-) vs 22% (SOX2+) whereas, using the SOX2/CDX2 molecular phenotypes, it becomes 41% (SOX2-/CDX2+) vs 14% (SOX2+/CDX2-).

**Figure 3:**
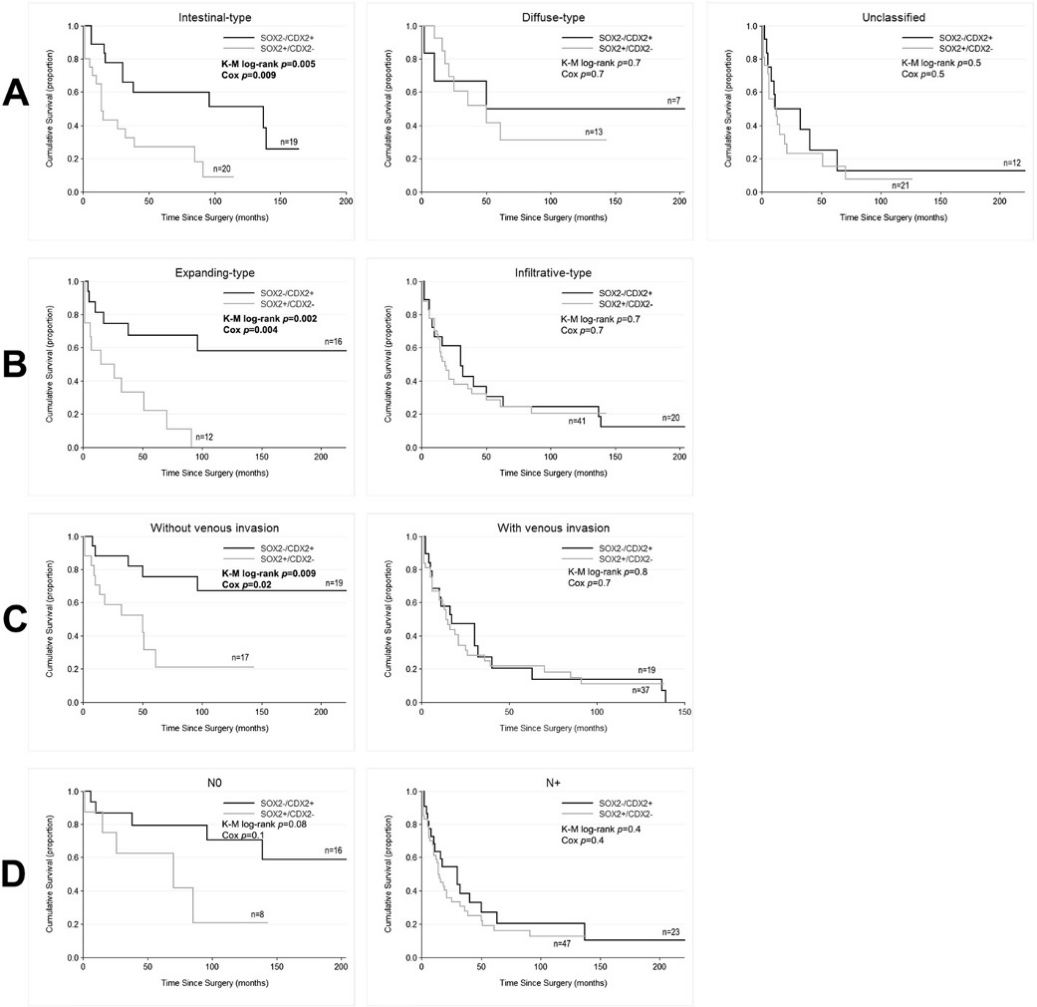
**Kaplan-Meier curves showing the probability of overall survival for patients with gastric cancer according to the molecular phenotypes SOX2** ^**-**^ **/CDX2** ^**+**^ **and SOX2** ^**+**^ **/CDX2** ^**-**^ **stratified according to clinicopathological parameters: Laurén classification (A), Ming classification (B), venous invasion (C) and lymph node metastases (D).**

We further explored the impact in patients’ survival of the molecular phenotypes defined by SOX2+ vs SOX2-, CDX2+ vs CDX2- and SOX2-/CDX2+ versus SOX2+/CDX2- stratified according to clinicopathological parameters. In this case, plotting survival according to either SOX2 or CDX2 status alone yielded significant prognostic differences for the former, in some of the parameters (intestinal- and expanding-type tumors) and no differences for CDX2 alone (Additional file 1: Figures S3 and S4). Results from Cox proportional model are presented in Additional file 1: Table S1. Once more, the use of the combined expression of SOX2 and CDX2 refined the previous results (Figure 3). SOX2^+^/CDX2^-^ profile predicted a significantly poorer outcome of patients with intestinal (*p* = 0.005) and expanding (*p* = 0.002) tumors, with hazard ratios of 3.18 and 4.84, respectively, and of those without signs of venous invasion (*p* = 0.009), presenting a hazard ratio of 3.84. Although borderline non-significant, a similar trend was observed in patients without lymph node metastases (*p* = 0.08) (Figure 3). Hazard ratios and 95% confidence intervals for all groups are presented in detail in Table 2 and remain significant after adjusting for age and gender in a multivariate analysis.

**Table 2:** **Cox Proportional Hazards Models of survival as a function of the molecular phenotypes (SOX2+/CDX2- vs SOX2-/CDX2+) for each clinicopathological parameter**

Parameter	Hazard ratio (95% CI)			
	Unadjusted	***P***	Adjusted ^a^	***P***
Overall	1.95 (1.15-3.31)	0.01	1.90 (1.10-3.28)	0.02
Laurén classification				
Intestinal-type	3.18 (1.33-7.58)	0.009	2.93 (1.21-7.10)	0.02
Diffuse-type	1.35 (0.35-5.17)	0.7		
Unclassified	1.34 (0.60-3.02)	0.5		
Ming classification				
Expanding	4.84 (1.64-14.29)	0.004	4.53 (1.29-15.85)	0.02
Infiltrative	1.13 (0.60-2.13)	0.7		
Vascular invasion				
Negative	3.84 (1.30-11.35)	0.02	3.82 (1.29-11.40)	0.02
Positive	1.10 (0.60-2.02)	0.8		
N stage				
N0	2.96 (0.82-10.65)	0.1	3.98 (0.72-21.83)	0.1
N+	1.29 (0.72-2.30)	0.4		

Abbreviation: *CI* confidence interval.

^a^ Adjusted for age and gender (Multivariate analysis using the Cox regression model).

### SOX2 amplification

Considering that gene amplification is a tumor-specific event during malignant transformation, and that SOX2 locus is subjected to this alteration in other tumors, namely of the lung and esophagus [17], we hypothesized that SOX2 amplification could explain its expression and heterogeneity, both within and among gastric tumors. Supporting this hypothesis, a survey performed on the Oncomine database showed that SOX2 ranked among the top 10% genes with copy number gains in gastric cancer (Additional file 1: Figure S5).

**Figure 4:**
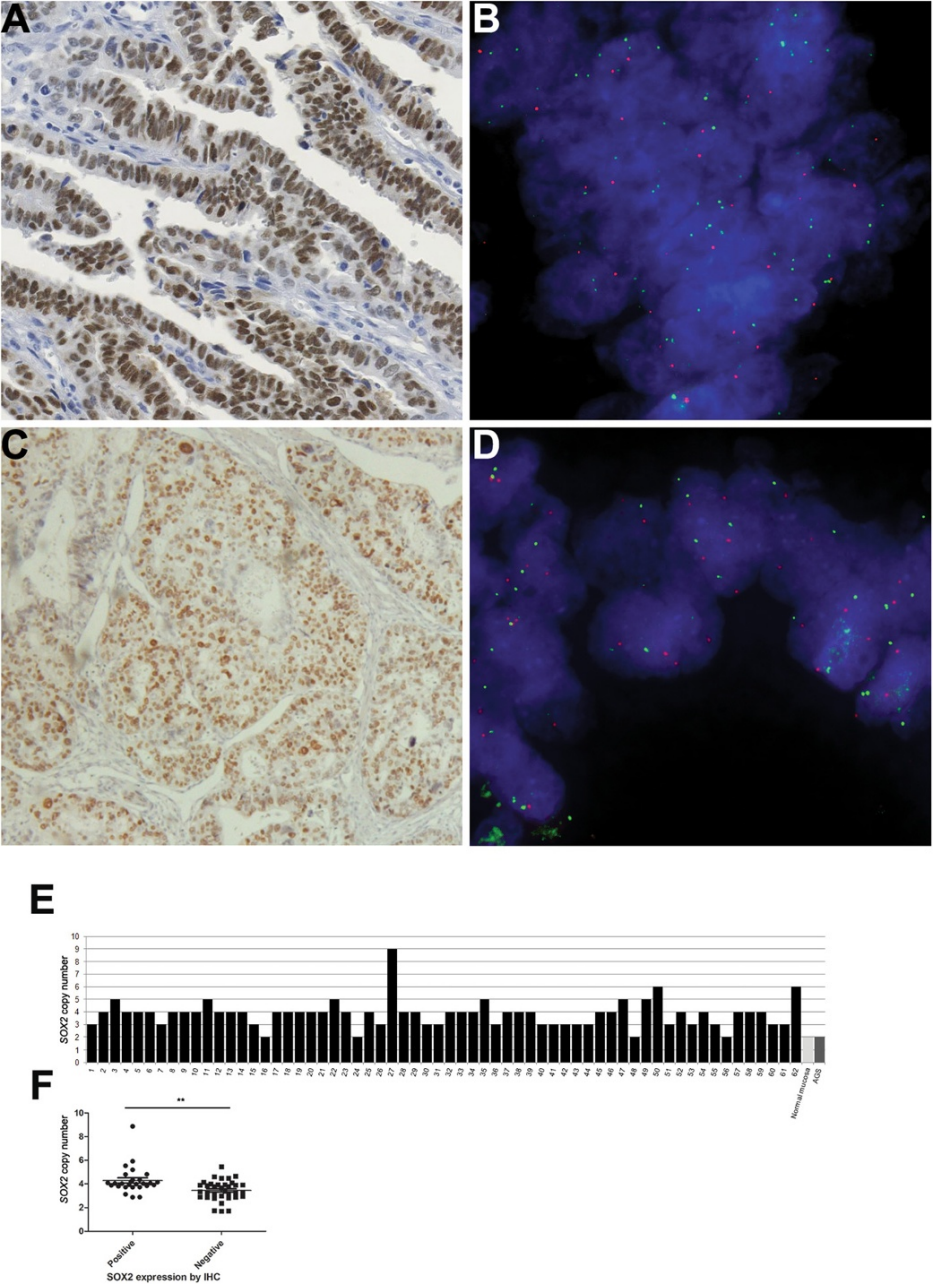
**Gastric cancer cases with SOX2 protein expression (A and C) can present with or without** ***SOX2*** **amplification (B and D, respectively) determined by FISH.** Green labeled probes target *SOX2*, whereas red labeled probes target the 3p arm. Nuclei are stained with DAPI (blue). **(E)** Sixty-two gDNA gastric cancer samples were assessed for SOX2 copy number status and compared to normal gastric mucosa (calibrator). **(F)** SOX2 copy number was significantly associated with protein expression, determined by IHC (*p* < 0.05).

Based on immunohistochemistry data, we selected 14 cases with SOX2 expression and 7 negative cases to analyse by 2-color FISH assay. We observed that SOX2 was amplified in 14% (2/14) of the cases with expression and in none of the cases without expression (0/7). A representative positive case both for SOX2 expression and amplification is shown in Figure 4A and B. A case with SOX2 expression but no amplification is shown in Figure 4C and D. Moreover, the FISH analysis demonstrated the presence of a high degree of copy number gain and heterogeneity within tumors, observed not only with the target probe for *SOX2* locus but also with the reference probe, located to the 3p arm (Additional file 1: Figure S6).

To clarify the frequency of *SOX2* locus copy number alterations in gastric cancer, we performed a copy-number PCR using gDNA from 62 gastric cancer cases, using a *RNAse P* probe located in chromosome 14 as reference. The majority of the samples (93%) presented some degree of *SOX2* copy number variation, ranging from 3 to 9 copies (Figure 4E). A significant positive association between copy number gain and SOX2 expression (*p* < 0.05) was observed (Figure 4F).

## Discussion

Gastric cancer is a disease with multiple outcomes that cannot be predicted by clinicopathological features alone [6]. Consequently, the identification of molecular biomarkers that may allow further outcome stratification and treatment decisions is of critical importance.

In this study we characterized the expression of the transcription factor SOX2 in gastric cancer and related it with clinicopathological features and differentiation markers namely MUC5AC (gastric marker) and CDX2 and MUC2 (intestinal markers). This led to the identification of two different prognostic groups, based on SOX2 expression, which could be further refined by taking into account the combined expression with CDX2. Finally, we unraveled the heterogeneity of *SOX2* locus and chromosome 3 copy number in gastric cancer, demonstrating an association between increased copy number and SOX2 protein expression.

A key finding of this study was the observation that SOX2 expression alone, and further refined by the combination with CDX2, allows the stratification of patients into subgroups with significantly different clinical outcomes. Moreover, when we combined these molecular profiles with clinicopathological data, it became evident that the SOX2^+^/CDX2^-^ profile associated with a poorer prognosis, within subsets of tumors that in general have a better prognosis: intestinal-type, expanding-type and with no signs of venous invasion [21, 22]. Although not statistically significant, the same tendency was observed for tumors presenting without lymph node metastases. These molecular profiles indicate a different biological behavior of the tumor most likely due to disease progression or treatment resistance and may be used to instruct more effective therapy or follow-up schedules.

Notably, as lymph node metastasis is one of the main risk factors for gastric cancer recurrence, after an R0 surgery, all patients presenting lymph node metastases receive adjuvant therapy [23]. However, our results show that patients without this risk factor, and thus not treated, present a strikingly worse outcome when the tumor presents with a SOX2^+^/CDX2^-^ molecular profile. This puts forward the hypothesis that, within N0 tumors, patients within this molecular subgroup would likely benefit from adjuvant therapy. Nevertheless, these observations require validation in larger cohorts and it would also be very informative to add clinical information regarding patient treatment.

SOX2 also emerges as a potential target for novel therapeutic interventions. Although transcription factors are not classical drug targets, approaches to SOX2-targeted therapy are already being addressed in breast cancer. In a recent study, *SOX2* gene was selectively targeted using zinc-finger-based artificial transcription factors approach, resulting in its highly specific, potent and long lasting down-regulation, translating into an attenuated tumorigenicity, both *in vitro* and *in vivo* [24].

Apart from the impact of SOX2 expression on patients’ survival, it is also associated with other clinicopathological features, namely male gender, more advanced T stage and venous invasion. The first is difficult to interpret but the last two suggest that SOX2 might have a role in the cellular properties that influence motility and invasion. Concordantly, in 13% of the cases SOX2 expression was higher in the tumor invasive front. This is in accordance with a study performed in a gastric cancer cell line [25], and other studies using different tumor models, namely gliomas, colorectal cancer and melanomas, where SOX2 was associated with disease progression and poor clinical outcome [26–28]. In the gastric cancer context, however, there are conflicting results with earlier reports suggesting a tumor suppressor function, based on *in vitro* studies [19] and loss of expression [18], not confirmed in more recent ones [20, 25], including ours.

Interestingly, SOX2 has been coined as a lineage-survival oncogene in different tumor models, proposing that cell lineage–specific genes involved in development can become dysregulated in cancer and promote tumorigenesis [17, 29]. SOX2 encodes a key stem cell transcription factor and additionally it is involved in the foregut morphogenesis [13]. In this study we did not find an association between SOX2 and gastric differentiation, evaluated by MUC5AC expression, which was previously described in gastric premalignant lesions and cancer [30, 31]. This observation, combined with the association with more aggressive clinicopathological features and worse survival, led us to hypothesize that, in this context, SOX2 may have other archetypal functions, associated to stemness features. These features would facilitate key processes such as epithelial to mesenchymal transition (EMT) and tumor spreading. This hypothesis is supported by numerous reports showing that SOX2 drives EMT in several cancer models, by upregulating several keystone proteins of this process, namely Snail, Slug and Twist [27, 32].

Another key observation in this study was the heterogeneity of SOX2 expression levels within and among cancers. Since SOX2 is a target of amplification in different tumors, we hypothesized that this genetic alteration, frequently associated with tumor progression, could explain not only the expression but also the heterogeneity of SOX2. The region 3q26-27, that encompasses *SOX2* locus, is a target of copy number gains in 18%-24% of gastric primary tumors [33]. Furthermore, copy number gain was also frequently detected in the long arm of chromosome 3 in gastric cell lines [34]. From the FISH results, combined with the copy-number PCR, which was performed in a larger set of gastric cancer cases, we can conclude that *SOX2* locus copy number gain is a frequent event in gastric cancer. Although genomic instability is found in most solid tumors and also in gastric cancer [35] it was surprising to find that more than 90% of the tumors present copy number gain of this locus. Notwithstanding, amplification of this gene, determined by FISH, could only be defined in 14% of the cases. Copy number gain is most likely not the sole mechanism associated with SOX2 expression since there are SOX2 positive cases without copy number gain and also the reverse. This was also observed in other tumor models, namely squamous carcinoma of the lung and gliomas [17, 26]. It remains to be confirmed whether, in the gastric context, SOX2 is the only target of copy number gain or amplification in this region. In fact, there are known oncogenes located in this genomic region [36] that might also be targets of copy number alterations and impact the biological behavior of the tumors. It has recently been shown that co-amplification of SOX2 and PRKCI contributes to the aggressive behavior of lung squamous cell carcinoma, by promoting a stem-like phenotype through activation of the Hedgehog signaling [37].

## Conclusions

In conclusion, we show for the first time that SOX2 together with CDX2 expression profiles in gastric cancer contributes to segregate patients into different prognostic groups, complementing the clinicopathological information. Moreover, we show that *SOX2* locus copy number gain is a frequent event in gastric cancer, explaining SOX2 protein expression and also pinpointing a genomic region frequently subjected to genomic instability.

## Electronic supplementary material

Additional file 1: Figure S1: Kaplan-Meyer curve showing the probability of overall survival for patients with gastric cancer. **Figure S2.** Kaplan-Meyer curves showing the probability of overall survival for patients with gastric cancer according to SOX2 and CDX2 combined expression profiles. **Figure S3.** Kaplan-Meyer curves showing the probability of overall survival for patients with gastric cancer according to SOX2 expression and stratified according to clinicopathological parameters: Laurén’s classification, Ming classification, venous invasion and lymph node metastasis. **Figure S4.** Kaplan-Meyer curves showing the probability of overall survival for patients with gastric cancer according to CDX2 expression and stratified according to clinicopathological parameters: Lauréns classification, Ming classification, venous invasion and lymph node metastasis. **Figure S5.** ONCOMINE gene microarray database was explored for SOX2 gene amplification and the results of The Cancer Genome Atlas (TCGA) for gastric cancer are displayed. **Figure S6.** Scatter plot showing the number of signals per cell for the *SOX2* locus and the *3p* arm, assessed by FISH. **Table S1.** Cox proportional hazards models of survival as a function of the SOX2 and CDX2 (positive *vs*. negative) for each clinicopathological parameter. (PDF 647 KB)
