# Supplementary material for: Immunohistochemical molecular phenotypes of gastric cancer based on SOX2 and CDX2 predict patient outcome
Source: BMC Cancer. 2014 Oct 9;14:753. doi: 10.1186/1471-2407-14-753 (PMC4210532; doi:10.1186/1471-2407-14-753)
Supplement: Supplementary file 1 — Additional file 1: Figure S1: Kaplan-Meyer curve showing the probability of overall survival for patients with gastric cancer. Figure S2. Kaplan-Meyer curves showing the probability of overall survival for patients with gastric cancer according to SOX2 and CDX2 combined expression profiles. Figure S3. Kaplan-Meyer curves showing the probability of overall survival for patients with gastric cancer according to SOX2 expression and stratified according to clinicopathological parameters: Laurén’s classification, Ming classification, venous invasion and lymph node metastasis. Figure S4. Kaplan-Meyer curves showing the probability of overall survival for patients with gastric cancer according to CDX2 expression and stratified according to clinicopathological parameters: Lauréns classification, Ming classification, venous invasion and lymph node metastasis. Figure S5. ONCOMINE gene microarray database was explored for SOX2 gene amplification and the results of The Cancer Genome Atlas (TCGA) for gastric cancer are displayed. Figure S6. Scatter plot showing the number of signals per cell for the SOX2 locus and the 3p arm, assessed by FISH. Table S1. Cox proportional hazards models of survival as a function of the SOX2 and CDX2 (positive vs. negative) for each clinicopathological parameter. (PDF 647 KB) [file 12885_2014_4943_MOESM1_ESM.pdf]

Supplementary data

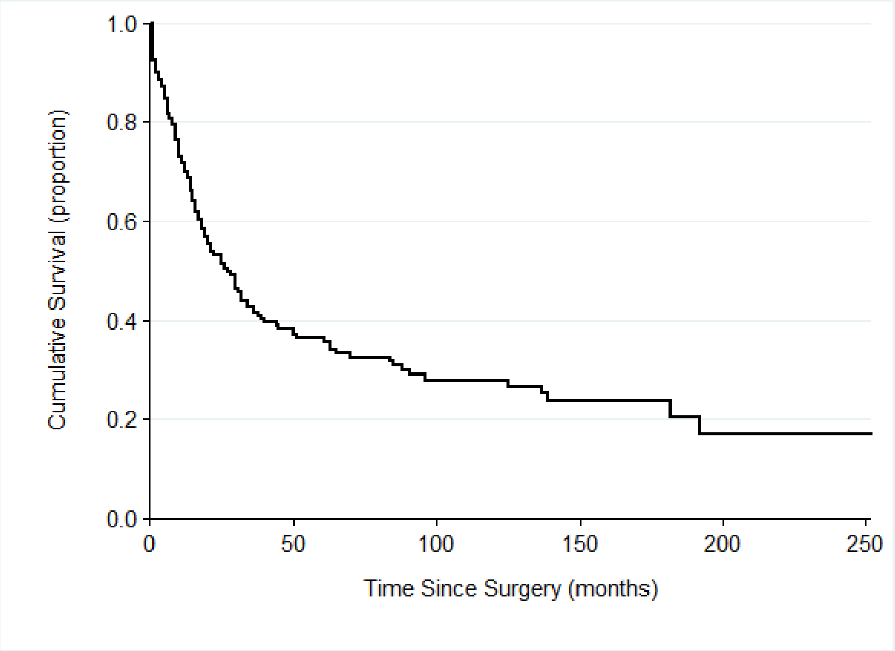

Supplementary Figure 1 - Kaplan-Meier curve showing the probability of overall survival for patients with gastric cancer.

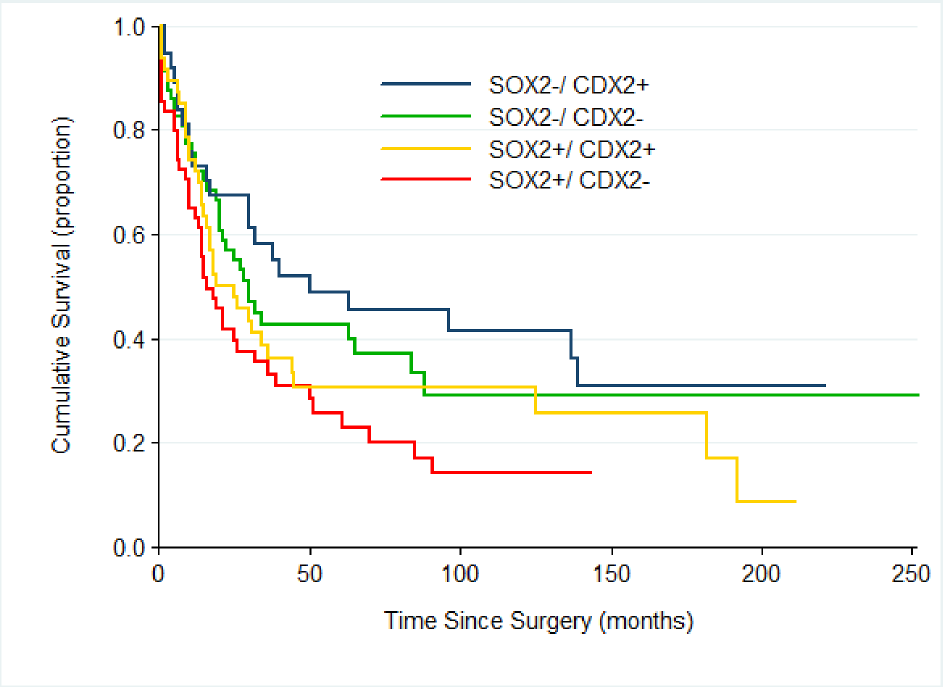

Supplementary Figure 2 - Kaplan-Meier curves showing the probability of overall survival for patients with gastric cancer according to SOX2 and CDX2 combined expression profiles.

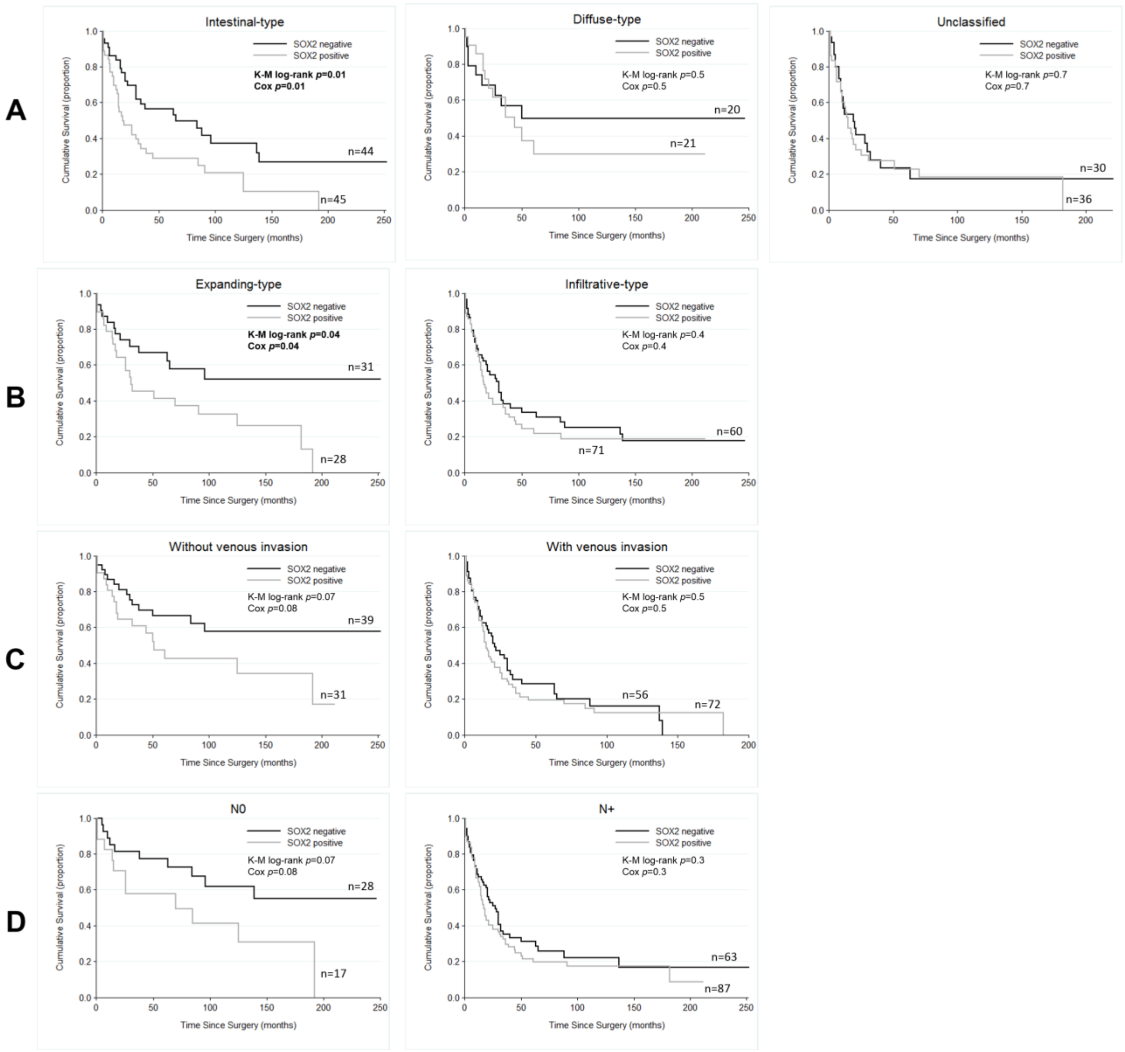

Supplementary Figure 3 - Kaplan-Meier curves showing the probability of overall survival for patients with gastric cancer, according to SOX2 expression, and stratified according to clinicopathological parameters: Laurén classification (A), Ming classification (B), venous invasion (C) and lymph node metastases (D).

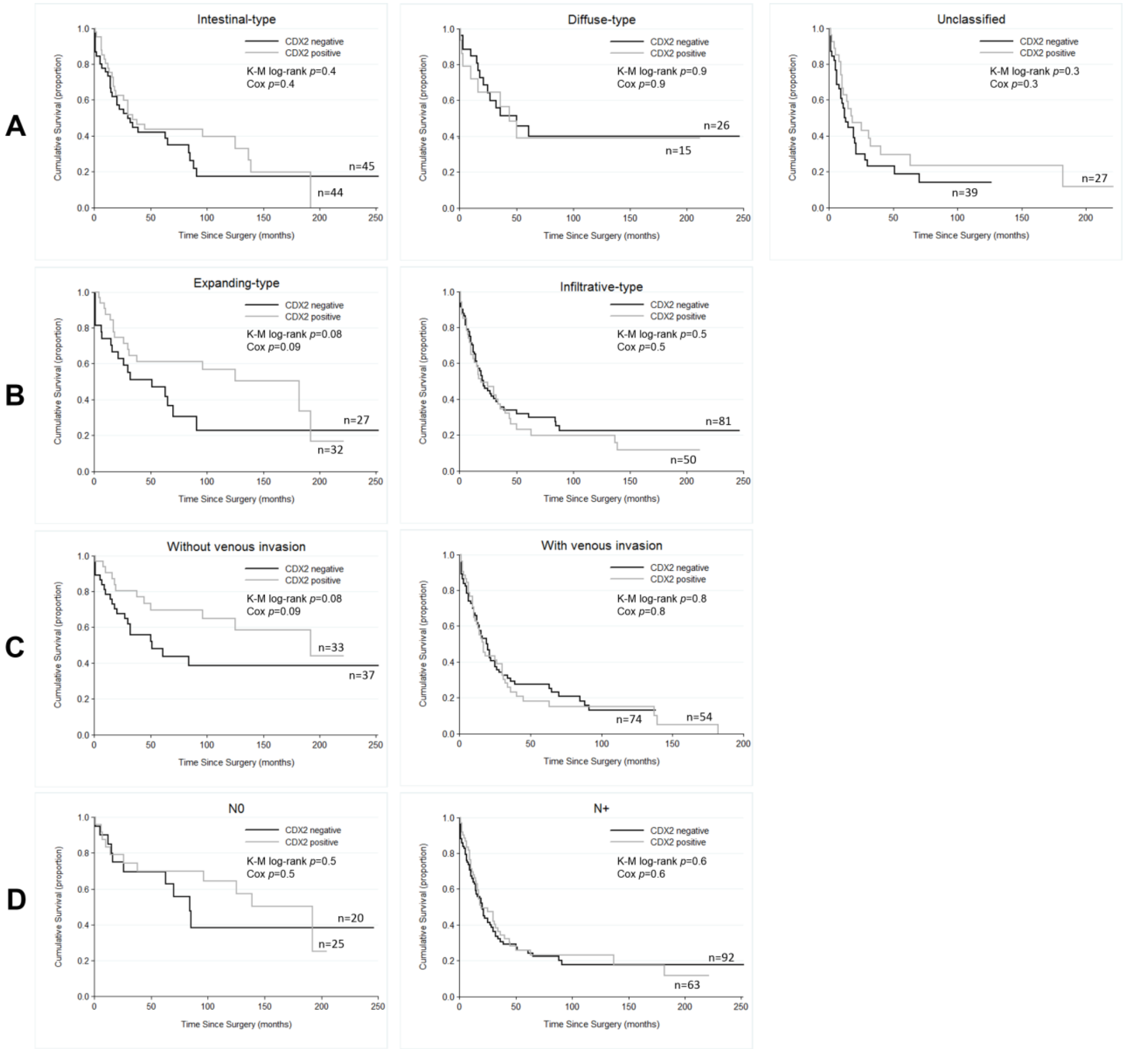

Supplementary Figure 4 - Kaplan-Meier curves showing the probability of overall survival for patients with gastric cancer, according to CDX2 expression, and stratified according to clinicopathological parameters: Laurén classification (A), Ming classification (B), venous invasion (C) and lymph node metastases (D).

Supplementary Table 1. Cox Proportional Hazards Models of survival as a function the SOX2 and CDX2 (positive vs. negative) for each clinicopathological parameter.

	SOX2		CDX2	
	HR (95% CI)	*P*	HR (95% CI)	*P*
Overall	1.51 (1.07-2.13)	0.02	0.82 (0.58-1.16)	0.3
Laurén classification				
Intestinal-type	1.94 (1.15-3.30)	0.01	0.79 (0.47-1.33)	0.4
Diffuse-type	1.31 (0.56-3.09)	0.5	1.07 (0.45-2.56)	0.9
Unclassified	1.19 (0.64-1.96)	0.7	0.72 (0.40-1.28)	0.3
Ming classification				
Expanding	2.05 (1.03-4.11)	0.04	0.55 (0.28-1.09)	0.09
Infiltrative	1.22 (0.80-1.84)	0.4	1.14 (0.75-1.73)	0.5
Vascular invasion				
Negative	1.89 (0.94-3.80)	0.08	0.54 (0.26-1.10)	0.09
Positive	1.16 (0.78-1.73)	0.5	1.05 (0.71-1.58)	0.8
N stage				
N0	2.15 (0.91-5.08)	0.08	0.76 (0.32-1.80)	0.5
N+	1.23 (0.85-1.79)	0.3	0.92 (0.63-1.34)	0.6

Abreviations: HR, hazard ratio; CI, confidence interval

SOX2 copy number in TCGA Gastric dataset

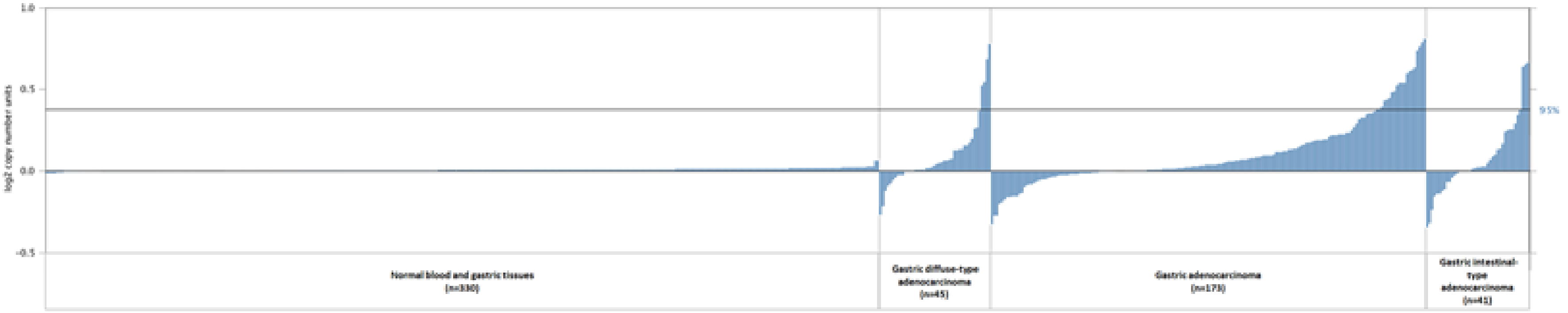

Supplementary Figure 5 – ONCOMINE gene microarray database was explored for SOX2 gene amplification and the results of The Cancer Genome Atlas (TCGA) for gastric cancers are displayed. This yielded a Cancer Outlier Profile Analysis (COPA) score at 32.595, ranking SOX2 in the top 10% copy number gain gene rank at 95th percentile among 18.823 measured genes.

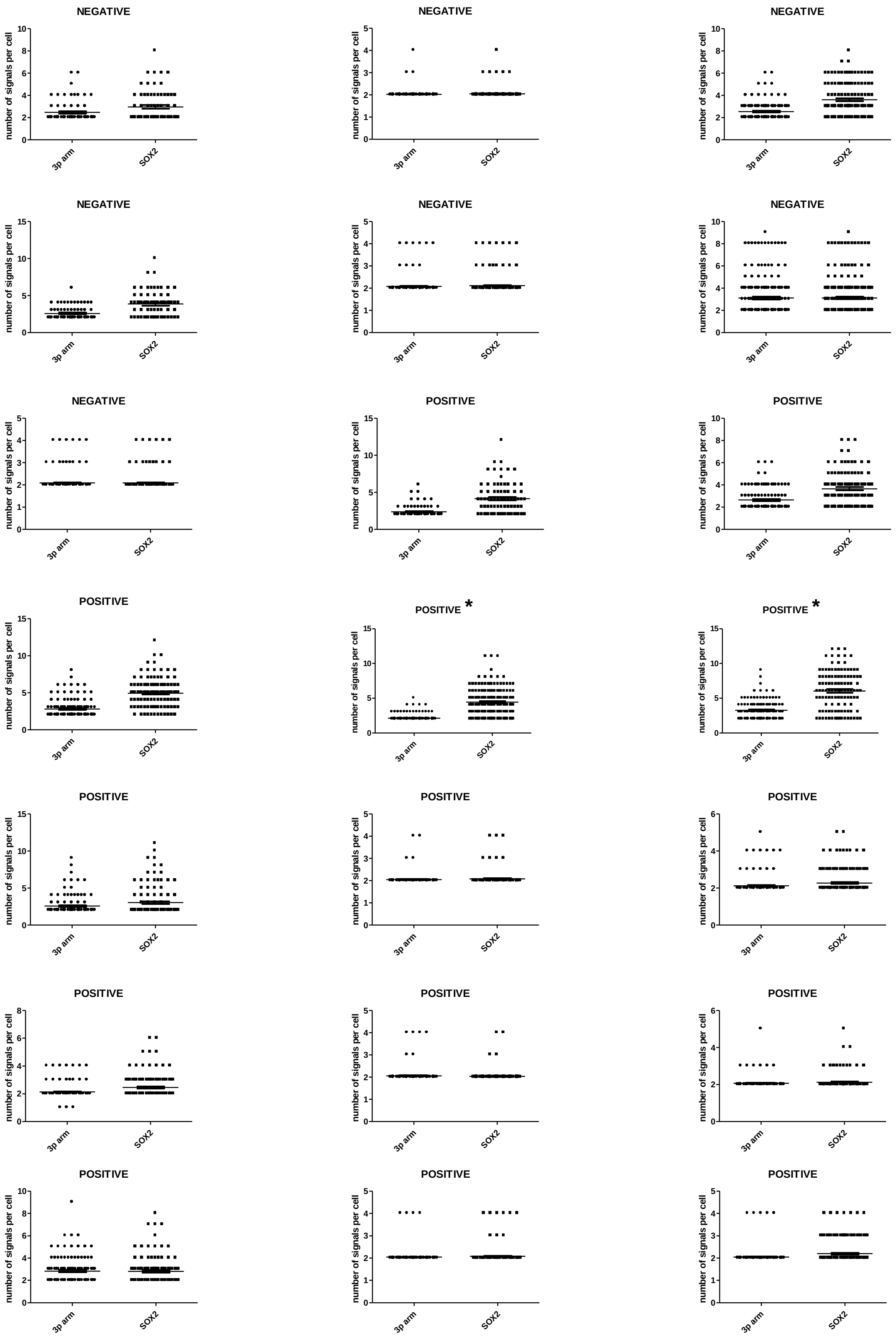

Supplementary Figure 6 – Scatter plot showing the number of signals per cell for both *SOX2* locus and the 3p arm, assessed by FISH. SOX2 IHC data is displayed on top.

* Case with amplification.
